# Comparison of a flexible versus rigid bone cement injection system in unilateral percutaneous vertebroplasty

**DOI:** 10.1186/s40001-020-00436-z

**Published:** 2020-08-25

**Authors:** Yuwei Li, Wei Cui, Peng Zhou, Cheng Li, Yan Wen, Wei Xiao

**Affiliations:** Department of Spinal Surgery, Luohe Central Hospital, Luohe Medical College, 56# Renmin Ave, Luohe, 462000 People’s Republic of China

**Keywords:** Osteoporosis, Percutaneous vertebroplasty, Osteoporotic vertebral fracture, Unilateral puncture

## Abstract

**Background:**

Percutaneous vertebroplasty (PVP) has been demonstrated to be effective in the treatment of acute osteoporotic vertebral fracture (AOVF). However, bilateral puncture takes more time to accept more X-ray irradiation; some scholars apply unilateral puncture PVP, but the cement cannot be symmetrically distributed in the vertebral body, so we use a flexible cement injector that undergoes PVP through the unilateral pedicle puncture. This research aims to compare the clinical results of PVP for AOVF with unilateral pedicle puncture using a straight bone cement injector and a bendable cement injector, determine the value of a bendable cement injector.

**Methods:**

We undertook a retrospective analysis of patients with thoracic and lumbar compression fracture treated with unilateral pedicle puncture percutaneous vertebroplasty from our institution from June 2013 to July 2015. Operation time, radiation exposure, bone cement injection amount, and the incidence of bone cement leakage were recorded on presentation, the cement leakage was measured by X-ray and computed tomography scan. The patients were followed up postoperatively and were assessed mainly with regard to clinical and radiological outcomes.

**Results:**

There was no significant difference in the operation time, radiation exposure time and incidence of bone cement leakage between the two groups. There was significant difference in the amount of bone cement injection and the difference between the two groups. There were no significant differences in VAS and the relative height of the vertebral body and local Cobb angle and QUALEFFO between the two groups at 1 week after PVP, significant difference was observed only 12 months after operation.

**Conclusions:**

Application of flexible cement injector is safe and feasible, compared with the application of straight bone cement injector, without prolonging the operative time, radiation exposure time and the incidence of bone cement leakage; it has the advantages of good long-term effect and low incidence of vertebral fracture recurrence.

## Background

Osteoporotic vertebral fracture is one of the most common diseases of the elderly [1–4]. Percutaneous vertebroplasty (PVP) is widely used to treat painful vertebral compression fractures and strengthen the stability of vertebrae [5, 6]. The usual standard in introduction of bone cement in PVP requires a bilateral pedicle approach to create a symmetrical distribution of bone cement [7]. However, pedicle puncture from both sides almost doubles the operation time and radiation exposure compared with a unilateral approach. Reducing operative time and radiation exposure is a valid objective. However, there is controversy about the efficacy of a unilateral approach. One study has reported similar short-term efficacy to bilateral procedures [8]. Another has suggested that introduction of bone cement unilaterally may lead to asymmetric loading of the vertebral body and collapse of the contralateral side of the vertebral body under axial compression stress [9].

## Materials and methods

The study protocol was approved by the Institutional Review Board and the Ethics Committee of Luohe Medical College.

### Patients

We undertook a retrospective analysis of patients with thoracic and lumbar compression fracture treated with unilateral pedicle puncture PVP from our institution over a 2-month period (June and July 2015). 78 patients were included according to the standard. Inclusion criteria includes: ① age from 60 to 99 years; ② bone attenuation (*T* score < − 2.5) on bone densitometry; ③ collapse more than 15% of the vertebral height; ④ severe back pain related to a single-level AOVF refractory to analgesic medication; ⑤ using magnetic resonance (MR) imaging, the affected vertebral body showed a hypointense signal on T1-weighted images and hyperintense signal on T2-weighted images. The exclusion criteria included: ① secondary osteoporosis (corticosteroids, endocrine disorders and an inflammatory process); ② failure to acquire informed consent; ③ uncorrected coagulopathy; ④ systemic or local spine infection; ⑤ painless AOVF; ⑥ spinal metastatic cancer; ⑦ severe comorbidities of the cardiorespiratory, hepatic, renal or neurological symptoms. Patients were divided into two groups according to surgical procedure: a flexible cement injection group (36 cases) using a flexible-tipped bone cement injection and 3-point cement injection technique; a rigid bone cement injection group (42 cases) using a straight bone cement injection technique.

### Surgical instruments

Flexible bone cement injection equipment (Ningbo Branch Huakerun Biotechnology Co., Ltd) with angled bone cement injector. Rigid bone cement injection equipment (Shandong Guanlong Medical Products Co., Ltd).

### Procedures

All the PVP procedures were performed in the operating theatre. Patients were placed prone, supported by two transverse bolsters under thorax and pelvis. Gentle distraction and extension was applied to reduce the vertebral fracture. During the procedure, a unilateral transverse process–pedicle approach was adopted with application of local anesthesia. The entry point in the vertebra was identified by fluoroscopy at the junction of the lateral edge of the pedicles and vertebral plate. The trocar penetrated cortical bone at the lateral edge margin of the vertebral arch, and was advanced medially and inferiorly. Fluoroscopy was used to confirm that the needle tip reached the posterior wall of the vertebral body. No further advance was made beyond about 4 mm anterior to the posterior surface of the vertebral body. During the procedure all patients were observed closely with frequent fluoroscopy and the cement injection was stopped immediately if bone cement leakage occured.

**Fig. 1:**
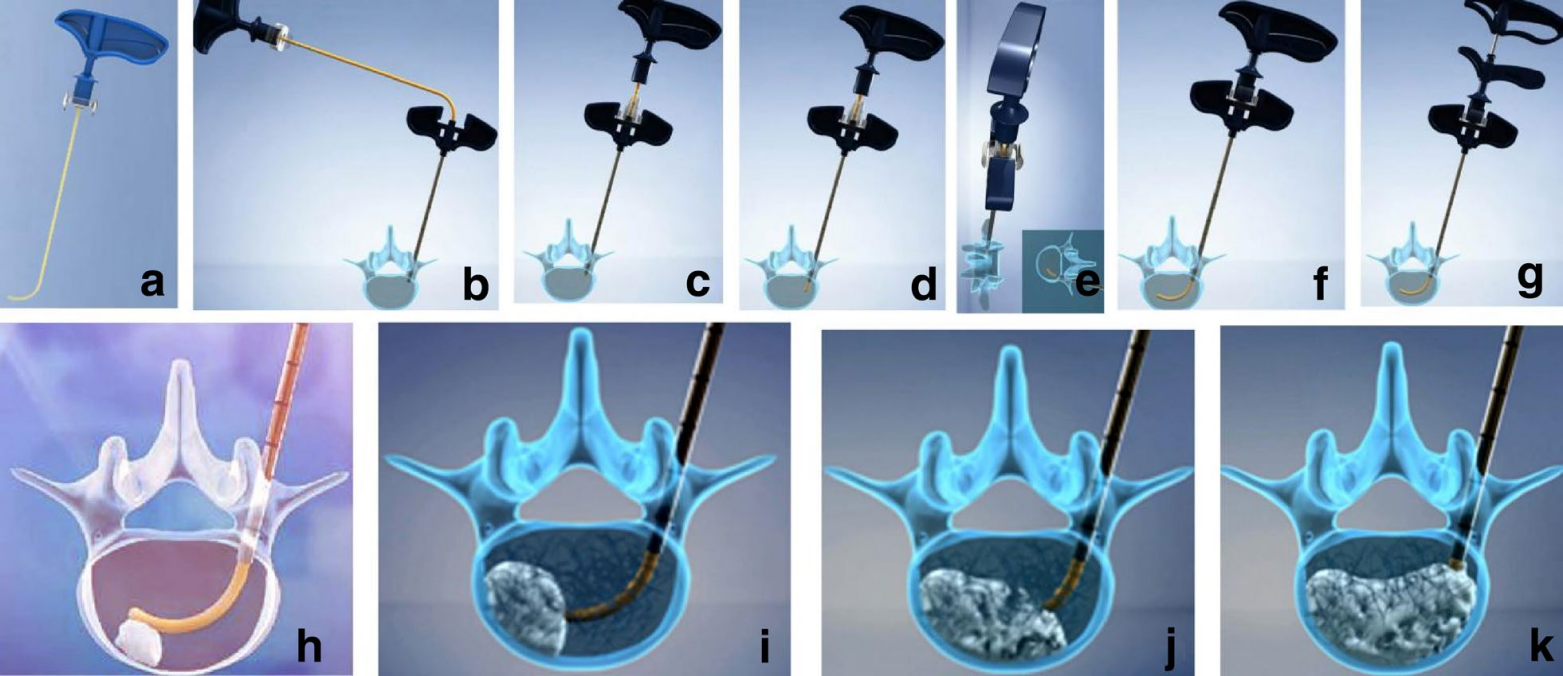
Diagrams of the flexible bone cement injection device and its mode of operation. **a** Flexible cement injector, showing its in-built curved tip. **b** Insertion of the flexible cement injector through the introducer. **c** A first mark on the flexible injector cannula identifies the point where the tip exits the introducer. **d** Using gentle back-and-forth rotation, the bone cement injector is advanced into the vertebral body. **e** On reaching the second mark on the flexible injection cannula, the tip of the cannula is located at the centre of the vertebral body. **f** When reaching the second scale at the end of the cement injector, it indicates that the cement injector head is on the opposite side of the vertebral body. **g** The central wire is removed. **h** The bone cement is first injected on the contralateral side of the vertebral body. **i** Approximately 2 ml of bone cement is used at this first point. **j** The injector is retracted to the second mark and a further 2-ml bone cement is injected. **k** The injector is retracted to the first mark and a further 2-ml bone cement is injected

**Fig. 2:**
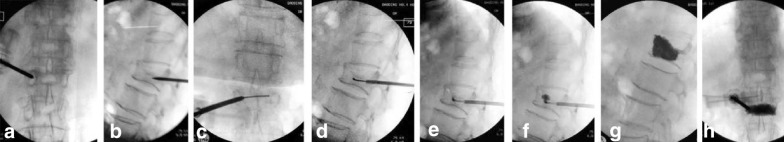
Intraoperative fluoroscopic images of the surgical procedure using the flexible injection system. **a** Posteroanterior fluoroscopic image: the trochar introducer penetrates the bone at the lateral edge of the pedicle. **b** Lateral fluoroscopy: the tip of the trochar reaches approximately 4 mm anterior to the posterior cortex of the vertebral body. **c**, **d** Posteroanterior and lateral fluoroscopies: the flexible cement injection cannula is directed towards the opposite side of the vertebral body. **e**, **f** Lateral fluoroscopies: bone cement is sequentially injected as the cannula is gradually retracted according to the steps described in Fig. 1. **g**, **h** Lateral and posteroanterior fluoroscopies: bone cement injection is complete

When using the rigid cement injector, an 11–13G core needle was advanced from a posterolateral entry point through the involved vertebral pedicle to the junction of anterior and middle thirds of the vertebral body. The inner core was retracted and 3–4 ml PMMA was injected guided by continuous fluoroscopy. When bone cement began to fill the posterior third of the vertebral body, the injection was terminated. In contrast, the flexible bone cement injection method is more involved; detailed descriptions of the process, can be found in the legends of Figs. 1 and 2.

After injection of bone cement, all injection components were withdrawn and pressure was applied to the wound for haemostasis. All the patients were observes supine for 6 h. The next-day rehabilitation included sitting and standing as tolerated. Bisphosphonates were generally used to treat osteoporosis after surgery.

### Outcome measures

The operation time, radiation exposure time, the amount of bone cement injection and the leakage of bone cement were recorded for each patient in two groups. Clinical assessments were evaluated before surgery, 1 week after surgery and 12 months after surgery. Radiographs and computed tomography (CT) scans were performed to assess the cement leakage in the vertebral body and other possible local complications, and all the complications and adverse events were recorded.

On pain measurement, VAS scores were used which ranged from 0 (no pain) to 10 (worst pain ever). Quality-adjusted life years (QALYs) and the Quality of Life Questionnaire of the European Foundation for Osteoporosis (QUALEFFO) were investigated in all patients, which comprise a 41-item questionnaire organized into 5 domains (Pain, Physical Function, Social Function, General Health Perception, and Mental Function). Each domain’s score and QUALEFFO total scores are recorded on a 100-point scale, lower scores corresponding to better health-related quality of life.

Anteroposterior and lateral standing radiographs were observed to measure vertebral height and kyphotic angle of the vertebral body of all patients in three periods (preoperatively, 1 week after surgery, and 12 months after surgery). In the X-ray radiographs, the anterior height of the affected vertebral body and adjacent normal vertebrae were measured, and the relative anterior height (RAH) of the fractured vertebra was calculated according to the equation:

RAH = fractured vertebral anterior height/[(superior vertebral anterior height + inferior vertebral anterior height)/2] × 100%.

**Fig. 3:**
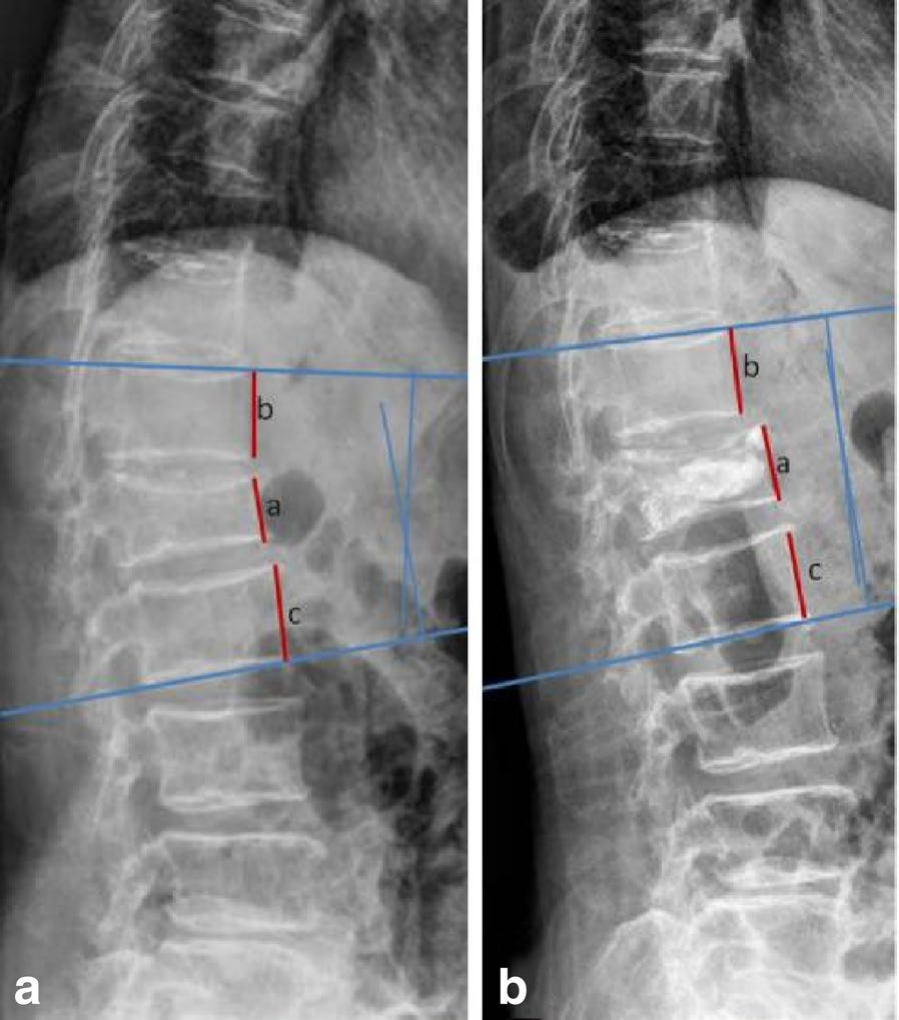
The relative anterior height of the fractured vertebra and the kyphotic angle measurement method. **a** TRA = a/[(b + c)/2]. **b** kyphotic angle = α

The kyphotic angle was based on the Phillips method, the angle between the superior endplate at one level above the fractured vertebrae and inferior endplate at one level below the fractured vertebrae were measured (Fig. 3).

### Statistical analysis

All statistical analyses were performed with the use of SPSS software, version 12 (SPSS Inc., Chicago, IL). The results were expressed as average ± SD. One-way analysis of variance (ANOVA) was used to compare the VAS scores, quality of life, RAH, and the kyphotic angle between the 2 groups. Difference in cement leakage rate of 2 groups was assessed using *χ*2 test. *P *< 0.05 was considered to have statistical significance.

## Results

**Fig. 4:**
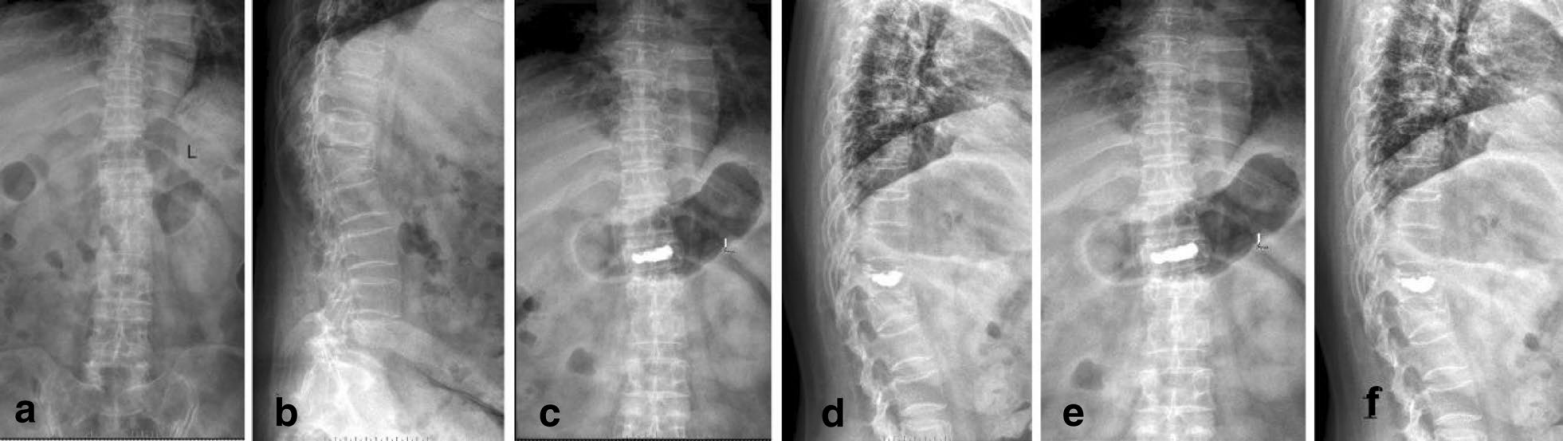
71-year-old woman with L2 vertebra fracture treated with flexible cement injector PVP. **a**, **b** Preoperative spinal column: L2 vertebral fractures. **c**, **d** 1 week after operation, spinal column: the distribution of bone cement is symmetrical. **e**, **f** Lumbar lateral position slice 2 years after operation: the bone cement remains symmetrically distributed, and the height of the injured vertebra has not changed

**Fig. 5:**
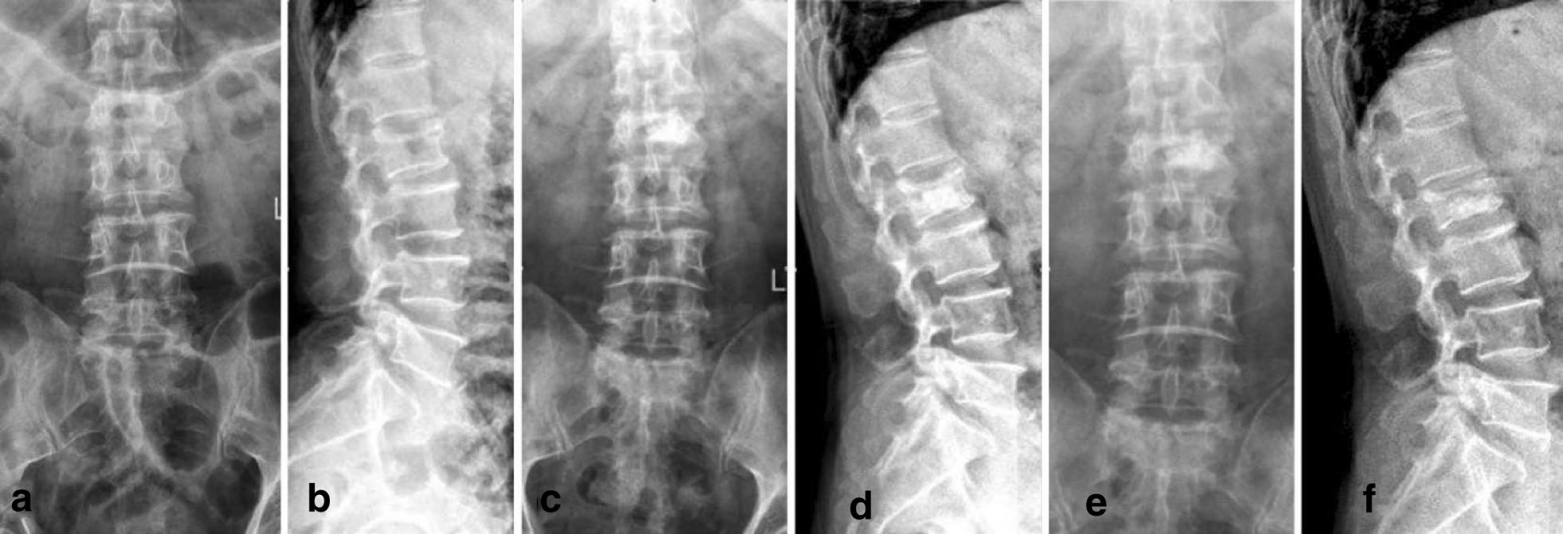
69-year-old woman with L2 vertebra fracture treated with straight bone cement injector PVP. **a**, **b** Preoperative spinal column: L2 vertebral fractures. **c**, **d** 1 week after operation, spinal column: bone cement is seen on one side of the vertebral body. **e**, **f** 2 years postoperative spinal column: vertebral height of the treated vertebra has diminished

All surgeries in two groups were completed successfully, and no intraoperative deaths were reported in this study. The average duration of follow-up was 15.8 months (range from 12 to 32 months). In terms of demographic data of patients, no significant difference was found between the two groups (Table 1). Typical cases are shown in Figs. 4, 5.

**Table 1:** Comparison of general data between treatment group and control group

Group	Number	Gender		Age, mean (years)	Body mass (kg)	Injury site		Bone density (SD)
		M	W			T	L	
Therapy group	36	20	16	67 ± 14	74 ± 10	22	14	−4.06 ± 0.33
Control group	42	24	18	66 ± 13	72 ± 12	24	18	−4.03 ± 0.31
Statistics		0.020		0.864	0.438	0.126		0.426
*P*		0.888		0.391	0.663	0.722		0.671

*T* thoracic, *L* lumbar

### Intraoperative measurement

There was no significant difference between two groups in the operation time and radiation exposure time (*P *> 0.05). The operation time was 28.4 ± 2.82 min (min) and 26.6 ± 2.35 min in the flexible cement injection (subject) group and rigid cement injection (control) group, respectively. In the flexible cement injection group, the radiation exposure time was 4.71 ± 0.95 min, compared with the radiation exposure time of 4.68 ± 0.80 min in the rigid cement injection group.

The average volume of the injected cement was 5.5 ± 0.35 ml and 3.2 ± 0.38 ml in flexible cement injection group and rigid bone cement injection group, respectively. A statistically significant difference was found between the two groups (*P* < 0.05) (Table 2).

**Table 2:** The two groups of operation time, radiation exposure time, bone cement injection, bone cement leakage contrast

Group	Operation time	Radiation exposure time	Bone cement injection	Bone cement leakage contrast
Therapy group	28.4 ± 2.82 min	4.71 ± 0.95 min	5.5 ± 0.35 ml	5 (13.9%)
Control group	26.6 ± 2.35 min	4.68 ± 0.80 min	3.2 ± 0.38 ml	6 (14.3%)
Statistics	*t *= 1.361	*t *= 0.138	*t *= 27.06	*x*^*2*^= 0.030
*P*	0.178	0.890	0.000	0.96

### Clinical results

No statistically significant differences were found in the baseline VAS and quality of life scores in the two groups. All scores were reduced in both groups after PVP surgeries, and there was no statistically significant difference between two groups at 1 week after PVP. However, statistically significant differences were found at 12 months after surgery (*P* < 0.05) (Table 3).

**Table 3:** Comparison of two groups of VAS and QUALEFFO

Group	Therapy group	Control group
VAS		
Preoperative	7.13 ± 044	7.16 ± 0.39
Postoperative 1 week	2.08 ± 011	2.11 ± 0.12
Postoperative 12 months	2.36 ± 0.15	4.5 ± 0.32
QUALEFFO		
Preoperative	60.88 ± 2.86	61.80 ± 2.83
Postoperative 1 week	41.45 ± 3.89	41.37 ± 3.56
Postoperative 12 months	36.88 ± 2.59	33.30 ± 1.61

### Radiological results

Preoperative and postoperative radiographical assessments of two groups were measured, and documented in Table 4. The RAH in the treatment group increased from 41.06 ± 5.58% preoperatively to 44.30 ± 3.80% at 1 week post-operation, and was 43.04 ± 5.19% 1 year postoperatively. In the control group, RAH increased from 43.01 ± 5.42% preoperatively to 45.49 ± 4.25% at 1 week post-operation, but was 30.86 ± 4.55% at 1 year post-surgery. There was no significant difference in RAH either preoperatively or 1 week postoperatively (*P *> 0.05). However, there were statistically significant differences between two groups after 12 months.

**Table 4:** Comparison of RAH and Cobb angles between two groups

Group	RAH (%)			Cobb (°)		
	Preoperative	1 week	1 year	Preoperative	1 week	1 year
Therapy group	41.06 ± 5.58	44.3 ± 3.8	43.04 ± 5.19	24.15 ± 4.07	22.68 ± 3.26	23.14 ± 3.53
Control group	43.01 ± 5.42	45.4 ± 4.25	30.86 ± 4.55	23.66 ± 3.35	22.60 ± 3.38	31.36 ± 5.41
Statistics	*t *= 1.566	*t *= 1.174	*t *= 11.04	*t *= 0.584	*t *= 0.108	*t *= 7.784
*P*	0.122	0.244	0.000	0.561	0.914	0.000

The Cobb angle in the treatment group changed from 24.15 ± 4.07° before surgery to 22.68 ± 3.26° at 1 week after surgery and 23.14 ± 3.53 at 1 year. In the control group it decreased from 23.66 ± 3.35° to 22.60 ± 3.38° at 1 week after operation, but had increased to 31.36 ± 5.41° at 1 year. There was no significant difference (*P *> 0.05) between two groups preoperatively and 1 week after surgery, however, a statistically significant difference was found between two groups after 12 months of follow-up, with the Cobb angle of the treatment group being smaller than that of the control group (Table 4).

### Complications

No procedure-related adverse events were observed in this study. Some extra-vertebral cement leakages were found in the intraoperative and postoperative radiographs. According to radiographic analysis, the rate of cement leakages was 13.9% (5 of 36) and 14.3% (6 of 42) in the therapy group and control group, respectively, and no significant difference was found between two groups (*P *> 0.05).

## Discussion

### Feasibility, safety and clinical efficacy of PVP with flexible cement injectors

Some studies showed that the relief of pain is associated with symmetrical distribution of bone cement in the vertebral body. The improvements of physical status largely depend on the volume of cement infusion during PVP [9, 10]; insufficient cement volume may lead to poor efficacy and failure of surgery. Conversely, increasing the amount of cement injection may increase the incidence of cement leakage [11–13]. Clinical studies have shown that 70% of complications of vertebral body forming are associated with the cement leakage [14–17]. To overcome the shortcomings of PVP technology in the area of sufficient and symmetrical cement distribution, the flexible bone cement injector was designed to deliver bone cement at three zones in the vertebral body. Due to the multi-point injection, the injected cement is in the state of low-pressure diffusion and subsequently bone cement leakage caused by high pressure has been avoided.

In traditional rigid injection cannulae, in order to distribute bone cement evenly on both sides of the vertebral body via the unilateral puncture, the angle of trocar approach should be as oblique as possible without damaging the medial cortex of the pedicle. This may risk neurological damage. This more oblique angle is not necessary in bilateral puncture, but the frequency of operative time and radiation exposure will be increased. These problems are overcome by using the flexible cement system [18].

In this study, the results confirmed the efficacy of PVP in the treatment of AOVF, which were reflected in immediate and significant change in VAS and QUALEFFO scores. Meanwhile, using flexible bone cement injector and three-region injection technology, it was shown that bone cement can be distributed more evenly in the vertebral body even punctured through single side of the vertebral pedicle.

### The influence of asymmetric parameters of vertebral body on PVP

We have not conducted in-depth study on the influence of preoperative vertebral asymmetric parameters on bone cement injection. The effect of PVP is related to the distribution of bone cement in the vertebral body. The ideal bone cement dispersion should be distributed on both sides of the midline of the vertebral body, with symmetrical distribution. If the asymmetric distribution of bone cement increases the transmission of unilateral load, the ideal effect cannot be achieved. There are many factors affecting the distribution of bone cement in vertebral body, including viscosity of bone cement, injection volume, puncture angle, vertebral body-related factors (bone density, vertebral structure, etc.)

### Long-term efficacy of the two systems

Murphy [19] reported that uneven distribution of the bone cement in the vertebral body may lead to further pressure-loading in the injury side of vertebra, and subsequent instability of spine. Under the constant loading, the vertebral body may buckle to the contralateral side, and thus causing further compression deformation of the vertebral body. The flexible bone cement injector cannula bends at its immediate exit from the introducer due to the elastic energy of the inner wire of Ni–Ti alloy, the curved angle channel reaches zones which a traditional rigid cannula cannot, thereby permitting a more uniform strengthening effect of the whole vertebra. In this study, compared with the control group, both vertebral height and back pain were significantly worse in the control group than the treatment group at 1-year follow-up. It seems likely that more uniform cement distribution and increased volume of cement improve the stability of the spine in the long-term.

There were some limitations in this retrospective study, and the sample of patients included is rather small. Moreover, follow-up periods in two groups were relatively short. Long-term follow-up data with a larger sample of patients are needed in future studies.

## Conclusion

This study confirmed that both flexible injector and straight bone injector PVP are safe and effective in the treatment of painful AOVF. During the period of follow-up (12 months), both methods showed good clinical outcomes. However, bone cement was distributed more evenly in the vertebral body via the flexible system without prolonging the surgical time, radiation exposure or increasing the incidence of bone cement leakage. The flexible cement injection technology demonstrated better long-term efficacy, compared with the traditional straight bone cement injection technology.

## Data Availability

All data generated or analysed during this study are included in this published article.

## Abbreviations

AOVF: Acute osteoporotic vertebral fracture
PMMA: Polymethyl methacrylate
PVP: Percutaneous vertebroplasty
QUALEFFO: Quality of life questionnaire of the European Osteoporosis Foundation
RAH: Relative anterior height of the fractured vertebra
